# Calcitonin gene-related peptide regulates spinal microglial activation through the histone H3 lysine 27 trimethylation via enhancer of zeste homolog-2 in rats with neuropathic pain

**DOI:** 10.1186/s12974-021-02168-1

**Published:** 2021-05-21

**Authors:** Qi An, Chenyan Sun, Ruidi Li, Shuhui Chen, Xinpei Gu, Shuhong An, Zhaojin Wang

**Affiliations:** Department of Human Anatomy, Shandong First Medical University & Shandong Academy of Medical Sciences, Taian, China

**Keywords:** Calcitonin gene-related peptide, Microglia, Histone H3 lysine 27 trimethylation, Neuropathic pain, ChIP-sequencing

## Abstract

**Background:**

Calcitonin gene-related peptide (CGRP) as a mediator of microglial activation at the transcriptional level may facilitate nociceptive signaling. Trimethylation of H3 lysine 27 (H3K27me3) by enhancer of zeste homolog 2 (EZH2) is an epigenetic mark that regulates inflammatory-related gene expression after peripheral nerve injury. In this study, we explored the relationship between CGRP and H3K27me3 in microglial activation after nerve injury, and elucidated the underlying mechanisms in the pathogenesis of chronic neuropathic pain.

**Methods:**

Microglial cells (BV2) were treated with CGRP and differentially enrichments of H3K27me3 on gene promoters were examined using ChIP-seq. A chronic constriction injury (CCI) rat model was used to evaluate the role of CGRP on microglial activation and EZH2/H3K27me3 signaling in CCI-induced neuropathic pain.

**Results:**

Overexpressions of EZH2 and H3K27me3 were confirmed in spinal microglia of CCI rats by immunofluorescence. CGRP treatment induced the increased of H3K27me3 expression in the spinal dorsal horn and cultured microglial cells (BV2) through EZH2. ChIP-seq data indicated that CGRP significantly altered H3K27me3 enrichments on gene promoters in microglia following CGRP treatment, including 173 gaining H3K27me3 and 75 losing this mark, which mostly enriched in regulation of cell growth, phagosome, and inflammation. qRT-PCR verified expressions of representative candidate genes (TRAF3IP2, BCL2L11, ITGAM, DAB2, NLRP12, WNT3, ADAM10) and real-time cell analysis (RTCA) verified microglial proliferation. Additionally, CGRP treatment and CCI increased expressions of ITGAM, ADAM10, MCP-1, and CX3CR1, key mediators of microglial activation in spinal dorsal horn and cultured microglial cells. Such increased effects induced by CCI were suppressed by CGRP antagonist and EZH2 inhibitor, which were concurrently associated with the attenuated mechanical and thermal hyperalgesia in CCI rats.

**Conclusion:**

Our findings highly indicate that CGRP is implicated in the genesis of neuropathic pain through regulating microglial activation via EZH2-mediated H3K27me3 in the spinal dorsal horn.

**Supplementary Information:**

The online version contains supplementary material available at 10.1186/s12974-021-02168-1.

## Introduction

Microglia are innate immune cells of the central nervous system that are responsible for the immoderate and chronic neuroinflammation following injury and disease [1]. Accumulating evidence suggests that microglia are involved in the development and maintenance of chronic neuropathic pain, as peripheral nerve injury of chronic pain models triggers microglial activation by induction of pro-inflammatory cytokine production in microglia [2, 3].

Recent studies suggest that epigenetic regulator is one of the most common causes in activation and suppression of various gene expressions in the persistent and development of chronic neuropathic pain models [4, 5]. Unfortunately, underlying mechanisms of gene expression alterations in the pathogenesis of neuropathic pain are not yet fully understood. Aberrant histone modifications, such as trimethylation of histone H3 at lysine-27 (H3K27me3) mediated, are closely associated with pro-inflammatory mediator expression in neuroinflammation [6]. It is well known that H3K27me3 modification at gene loci represses gene transcription [7, 8]. Recent study indicated that enhancer of zeste homolog 2 (EZH2, the histone methyltransferase of polycomb repressive complex 2)-mediated H3K27me3 has been recognized to play a critical role in the regulation of activation of microglia and production of proinflammatory mediators in the development of neuropathic pain [8].

Calcitonin gene-related peptide (CGRP) as a mediator of microglial activation, may facilitate nociceptive signaling through action on microglial CGRP receptors and release of ATP in dorsal horn [9, 10]. CGRP receptor components that consist of calcitonin receptor-like receptor (CRLR), receptor activity-modifying protein 1 (RAMP1), and receptor component protein (CRCP) were expressed in activated microglial cells in neuroinflammatory disease [11]. It has been reported that CGRP induced the activation of microglia at the transcriptional level through expression of the immediate-early genes c-fos in the spinal cord [10, 12], suggesting that CGRP may play a physiological role as a regulator of microglial gene expression. Our previous research showed that CGRP is involved in the expression of immune and inflammation-related genes in microglia through epigenetic mechanism [13].

The ability of CGRP to activate microglia raises the question of whether the inflammatory gene expression induced by CGRP associates with the EZH2/H3K27me3-mediated pathophysiology of neuropathic pain [12, 14]. Therefore, the present study was carried out to compare the different H3K27me3 enrichment profiles of microglia treated with CGRP and controls using chromatin immunoprecipitation sequencing (ChIP-seq) to gain a better understanding of a potential role for this peptide in the activation of microglia. The effect of CGRP on the expression of EZH2 and H3K27me3 in the spinal dorsal horn and the genesis of neuropathic pain were also examined in the chronic constriction injury (CCI) rat model, hoping that these studies could further understand the underlying regulatory mechanism of microglia by CGRP in neuropathic pain pathophysiology at the molecular level.

## Methods

### Animals and CCI rat model

Adult male Wistar rats weighing 200250 g were obtained from the Animal Center of Shandong First Medical University. All experimental procedures followed the guidelines of the Shandong First Medical University Institutional Animal Care and Use Committee (Approval No. 2018025). CCI to the sciatic nerve of the right hind limb in rats was performed based on previous description [15]. Briefly, animals were anesthetized with isoflurane (1.5%). The sciatic nerve of the right hind limb was exposed at the middle of the thigh by blunt dissection. To prevent the interruption of blood circulation through the epineural vasculature, four chromic gut ligatures were loosely tied (4.0 silk) around the nerve with spacing at ~ 1 mm. In the control group, the right sciatic nerve was exposed for 23 min, but was not ligated. Following surgery, the skin was closed with a single suture, and animals were allowed to recover for 14 days. All behavioral tests were performed by mechanical withdrawal threshold (MWT) and thermal withdrawal latency (TWL). Mechanical allodynia and thermal hyperalgesia are reproducible and sensitive behavioral readouts of neuropathic pain.

### Intrathecal implantation

Intrathecal implantation was performed as described previously [16, 17] by inserting polyethylene tubing through which the drug was directly injected into the subarachnoid space of the lumbar enlargement. After surgery, neurologically normal rats were injected with 2% lidocaine (10 L) through the intrathecal catheter to confirm that the polyethylene tubing was in the subarachnoid space. Only those rats showing complete paralysis of both hind limbs after the administration of lidocaine were used for the subsequent experiments. Animals with the intrathecal catheter were then randomly divided into CCI and sham operation, respectively. The CGRP (1 M, Tocris Bioscience), GSK126 (EZH2 inhibitor, 5nM, MEC), CGRP8-37 (CGRP antagonist, 2 M, MCE) or vehicle in a volume of 10 L was injected into the spinal lumbar enlargement region through the intrathecal catheter, followed by 20 L of saline to flush. Previous studies have demonstrated that these dosages of CGRP, GSK126, CGRP8-37, and other reagents in experiments proved to be effective in vivo and in vitro [8, 9, 13, 18, 19]. When the drug administration fell on the same day as the behavior analysis, behavior tests were completed prior to the drug administration. At the end of each experiment, the position of the polyethylene tubing in the intrathecal space at the lumbar enlargement was visually verified by exposing the lumbar spinal cord. Data from rats with incorrect polyethylene tubing position were discarded from the study.

### Cell culture and drug administration

BV2 microglial cells those are positive for differentiated microglial markers (eg., CD11b, CD45, Iba1, TMEM119) [20] was obtained from the Cell Bank of the Chinese Academy of Sciences (Beijing, China). Cells were cultured in DMEM supplemented with 10% fetal bovine serum (FBS, Biological Industries) incubated at 37 C in an atmosphere of 5% CO_2_. BV2 cells continuously stimulated with CGRP peptide (1 M,) at 0, 1, 2, 4, 6, and 12 h, respectively. Cells without CGRP peptide were used as control. To assess the possible underlying signaling pathways for the effect of CGRP, 1M forskolin (cAMP/PKA activator), 3 M myristoylated PKA inhibitor fragment 6-22 (PKI6-22, PKA inhibitor, RD), 325 nM phorbol 12-myristate 13-acetate (PMA, PKC activator, MEC), 5 M chelerythrine chloride (PKC inhibitor), or 5 M GSK126 were preapplied for 30 min and coapplied together with CGRP for 4 h at 37 C.

### Isolation and characterization of rat primary microglia

Rat primary microglia were isolated and characterized as previously described [21]. Cerebral cortices of 12-day-old Wistar rats were surgically removed, placed in cold DMEM, and brain tissue minced and dissociated with trypsin-EDTA at 36 C for 35 min. The mixed glial cell suspension was plated in vented cell culture flasks with DMEM medium supplemented with 10% FBS, and grown in a humidified 5% CO_2_ incubator at 36 C for 1214 days. Upon confluence (day 14) and every week thereafter, microglia were detached using an orbital shaker (150 rpm, 0.5 h, 36 C, 5% CO_2_), centrifuged (400 *g*, 25 min, 4 C), and cell number and viability were assessed by trypan blue exclusion. Purified microglia obtained by this method averaged more than 95% viability.

### Immunofluorescence

Animals were perfused through the ascending aorta with 100150 ml saline followed by 300 ml 4% paraformaldehyde in 0.1 M phosphate buffer (pH 7.4). L4L5 spinal cord segments were removed, postfixed in the same fixative for 4 h at 4C and cryoprotected in 20% sucrose overnight. Transverse 8-m-thick sections were cut on a cryostat and processed for immunofluorescence. In order to reveal the coexistence of either EZH2 or H3K27me3 with Iba1 (a marker for microglia) or NeuN (a marker for neurons) double immunostaining on the same sections was used. Sections were incubated with primary antibodies against CGRP (Merck Millipore), EZH2 (CST), and H3K27me3 (Abcam) with Iba1 (Abcam) or NeuN (Abcam) overnight at room temperature. Following three washes with tris-buffered saline (TBS), sections were treated with a 1:1 mixture of the matching FITC- and Cy3-conjugated secondary antibodies (Jackson Immunoresearch). After washing three times in TBS, sections were counterstained with DAPI (Abcam). The specificity of antibodies used was checked by western blotting and/or omission of the primary antibodies. No specific immunoreactivity was detected in these tissue sections.

### Quantification of immunofluorescence

Quantitative analyses of the percentage of immunostaining surface in the spinal cord laminae III (CGRP) and the whole spinal dorsal horn (Iba1) were conducted with Image Pro-Plus program as described previously [17]. Briefly, the background in pictures was first subtracted with a uniform standard. The regions for laminae III and the whole spinal dorsal horn in the spinal sections were artificially selected. Then, the threshold values of fluorescent intensity for positive immunoreactivity were set, and the percentage of immunostaining areas were obtained by the Image Pro-Plus program. The numbers of EZH2- and H3K27me3-positive microglia or neurons in the spinal dorsal horn were counted. For each animal, the data from five different rostrocaudal planes within L4 and L5 spinal cord segments was obtained, and six animals in each group were evaluated to get the mean values.

### Immunofluorescence of cultured microglial cells

Mouse microglial cells (BV2) and rat primary microglia were cultured on poly-L-lysine-coated coverslips. Following a single wash in phosphate buffered saline (PBS), cultured microglial cells were fixed in 4% paraformaldehyde for 15 min at room temperature. Double-labeling immunofluorescence staining for primary antibodies against Iba1 and CRLR (Abcam), RAMP1 (Sigma-Aldrich), or CRCP (Proteintech) on coverslip-cultured microglial cells was performed. Coverslips were incubated with a mixture of the two primary antibodies overnight. Coverslips were then incubated with FITC- and Cy3-conjugated secondary antibodies (Jackson Immunoresearch). After washing three times in TBS, coverslips were counterstained with DAPI (Abcam).

### Western blotting

Cultured microglial cells or the dorsal quadrant of L4L5 spinal segment ipsilateral to the operation side were lysed, and the protein was extracted. The protein lysate from each sample was separated electrophoretically on a sodium dodecyl sulfate-polyacrylamide gel and then transferred to a polyvinylidene fluoride (PVDF) membrane. After blocking with 5% nonfat milk in TBS-T (containing 0.1% Tween-20) for 2 h, membranes were incubated with primary antibodies against CGRP (Absin), EZH2, H3K27me3, ITGAM (CR3, Abcam), ADAM10 (Abcam), MCP-1 (Abcam), and CX3CR1 (CST) in 5% nonfat milk in TBS-T overnight at 4 C. After washes with TBS-T, membranes were incubated with the appropriate secondary antibodies for 2 h. Results were visualized using an ECL chemiluminescence system. GAPDH antibody (CST) was also used as a probed control to ensure the loading of equivalent amounts of the sample proteins. The band densities were compared in TotalLab software (version 2.01; Bio-Rad, Hercules, CA).

### Real-time cell analysis (RTCA)

Microglial cells were seeded at 10^3^ cells/well in 96-well E-plates (Roche) with an integrated microelectronic sensor array in 100 L of suitable culture medium (RTCA DP, ACEA Biosciences). After 24 h, 5 M GSK126 was coapplied together with 1 M CGRP to a total volume of 100 L. Cell proliferation and viability were monitored in real-time by measuring the cell-to-electrode responses of the seeded cells. The cell index (CI) was calculated for each E-plate well by RTCA Software. The graphs are generated in real time by the xCELLigence system.

### Chromatin immunoprecipitation

Chromatin was prepared from fixed mouse microglial cells (stimulated with 1 mol/L CGRP, 4 h) and sonicated fragments ranged in size from 200 to 1500 bp. Approximately 2 10^7^ cell equivalents were used for each immunoprecipitation. ChIP was performed as described previously [22], using anti-H3K27me3 antibody (ChIP Grade, ab6002, Abcam), or a control rabbit IgG.

### Sequencing library preparation, cluster generation, and sequencing

DNA samples were end-repaired, A-tailed, and adaptor-ligated using TruSeq Nano DNA Sample Prep Kit (#FC-121-4002, Illumina), following the manufacturers instructions. Approximately 200 to 1500 bp fragments were size selected using AMPure XP beads. The final size of the library was confirmed by Agilent 2100 Bioanalyzer. The samples were diluted to a final concentration of 8 pmol/L, and cluster generation was performed on the Illumina cBot using HiSeq 3000/4000 PE Cluster Kit (#PE-410-1001, Illumina), following manufacturers instructions. Sequencing was performed on Illumina HiSeq 4000 using HiSeq 3000/4000 SBS Kit (300 cycles) (#FC-410-1003, Illumina), according to the manufacturers instructions.

### Data collection and ChIP-seq analysis

After the sequencing platform generated the sequencing images, the stages of image analysis and base calling were performed using Off-Line Basecaller software (OLB V1.8). Sequence quality was examined using the FastQC software. After passing Solexa CHASTITY quality filter, clean reads were aligned to mouse genome (UCSC MM10) using BOWTIE software (V2.1.0). Aligned reads were used for peak calling of the ChIP regions using MACS V1.4.2. Statistically significant ChIP-enriched regions (peaks) were identified by comparison of IP vs Input or comparison to a Poisson background model, using a *p* value threshold of 10^-4^. The nearest gene using the newest UCSC RefSeq database annotated peaks in samples. The annotation of the peaks, which were located within 5 kb to + 5 kb around the corresponding gene across the transcription start sites (TSSs) in samples, can be found from the peakpromoter annotation.

### Bioinformatics analysis

The Gene Ontology (GO) functional and Kyoto Encyclopedia of Genes and Genomes (KEGG) pathway enrichment analysis were performed using the Database for Annotation, Visualization and Integrated Discovery (DAVID) and KEGG Orthology-Based Annotation System (KOBAS) online tools (https://www.geneontology.org and https://www.genome.jp/kegg).

### RNA extraction and quantitative real-time PCR

The expression profiles of genes selected from enriched GO terms that derived from ChIP-seq data were assessed by qRT-PCR at 4 h after treatment of CGRP with microglial cells. The expression of GAPDH mRNA was also determined as an internal control. Total RNA was isolated from cultured microglial cells using Trizol reagent (Invitrogen) according to the manufacturers protocol. RNA concentration was determined spectrophotometrically. After this, cDNA was synthesized using a cDNA synthesis kit (Invitrogen) according to the manufacturers instructions. Primer sequences are listed in the Supplementary Table S1. qRT-PCR was performed in triplicates by using a 7300 real-time PCR system (Applied Biosystems, Foster City, CA) according to the manufacturers instructions. A comparative cycle of threshold fluorescence (Ct) method was used, and the relative transcript amount of target gene was normalized to that of GAPDH using the 2^-Ct^ method. The results of qRTPCR were expressed as the ratio of test mRNA to control.

### Statistical analysis

All experiments were independently repeated at least three times. Data are presented as the meansSEM. MannWhitney *U* tests were used for comparisons between two groups, and KruskalWallis tests with Dunns multiple comparisons post hoc tests were used for comparisons among multiple groups. The MWT or TWL among groups were analyzed by two-way repeated measures ANOVA with groups and time points as independent factors, followed by Bonferroni post hoc tests. Significance was set at *p* < 0.05.

## Results

### Model identification of neuropathic pain

**Fig. 1:**
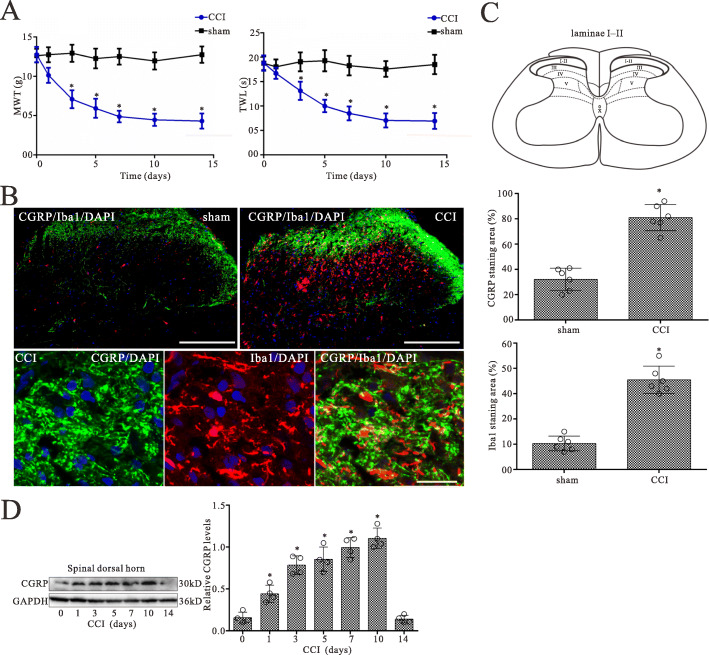
CCI evokes increases in the expressions of CGRP and Iba1 in the L4L5 spinal dorsal horn of CCI rats. **a** Nociceptive behavior developed in CCI model rats. Mechanical withdrawal threshold (MWT) and thermal withdrawal latency (TWL) were examined at 0, 1, 3, 5, 7, 10, and 14 days respectively after sham operation or CCI surgery. *n* = 6, ^*^p < 0.05 vs. sham group. **b** Double-staining immunofluorescent images showing the CGRP-positive fibers (green) and Iba1 (microglia maker)-positive microglia (red) in the dorsal horn of sham and CCI groups on day 5 after surgery. Cell nuclei were stained with the DAPI (blue). Note that numerous varicose nerve terminals immunoreactive for CGRP (green) closely approached and surrounded Iba1 immunopositive microglia (red) in the laminae I and II of the spinal dorsal horn (second row). Scale bar of 200 m in the first row and 10 m in the second row. **c** Quantitative analysis of the percentages of CGRP-immunoreactive surface in laminae I and II, and Iba1-immunostaining surface in the spinal dorsal horn showed the CCI-induced changes. Data are presented as the mean SEM (*n* = 6). ^*^p < 0.05 vs. sham group. **d** Western blot analysis of CGRP expression in the spinal dorsal horn on 0, 1, 3, 5, 7, 10, and 14 days after CCI surgery, respectively. The mean optic density of the protein was calculated by normalizing to GAPDH. All values are expressed as the means SEMs (*n* = 4).^*^p < 0.05 vs. sham group

To assess the chronic pain status induced by CCI model, both mechanical allodynia and thermal sensitivity of animal hind paws were evaluated at 0, 1, 3, 5, 7, and 14 days after surgery, respectively. MWT and TWL of CCI-ipsilateral hind paws were significantly lower than those of both sham-ipsilateral on postoperative days 3 to 14 and reached a steady peak at day 14 after surgery (Fig. 1a), indicating CCI induced mechanical allodynia and thermal sensitivity of hind paws.

### CCI evokes increase in CGRP- and Iba1-immunoreactivity in the spinal dorsal horn

In the control group, CGRP immunoreactivity was confined to superficial laminae, mainly in laminae III which is the main region involved in pain process in the dorsal horn, and some sparse CGRP-labeled fibers were present in laminae IIIIV (Fig. 1b). On day 5 after surgery, CGRP immunofluorescence intensity was significantly increased in the superficial laminae III in the ipsilateral L4L5 spinal dorsal horn (Fig. 1b, c). Western blot data showed that CCI evoked significant increase in CGRP protein expression on postoperative days 1, 3, 5, 7, 10, and 14 respectively (Fig. 1d). The largest increase in expression for these time points was seen on day 10 after injury. A recovery of CGRP expression was seen on postoperative day 14.

Increased expression of Iba1 represents microglial activation during nerve injury [23]. To investigate the mechanisms by which CCI induces neuropathic pain, the expression level of Iba1 was then detected by immunofluorescence at 5 days after injury. Immunofluorescent staining analysis showed significant enhancement of Iba1 expression in the L4L5 spinal dorsal horn on postoperative day 5 (Fig. 1b, c). Double immunofluorescence revealed that numerous varicose nerve terminals immunoreactive for CGRP closely approached and surrounded Iba1 immunopositive microglia in the ipsilateral spinal dorsal horn (Fig. 1b).

### CCI induced increase of EZH2 and H3K27me3 expressions in the spinal dorsal horn

**Fig. 2:**
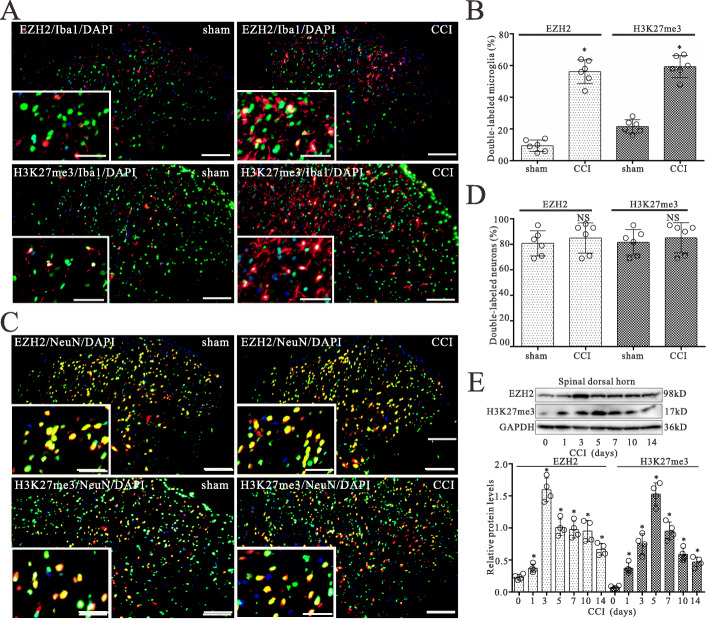
CCI evokes increases in the expressions of EZH2 and H3K27me3 in the L4L5 spinal dorsal cord of CCI rats. **a** Double-staining immunofluorescent images showing the expression of EZH2 (green) and H3K27me3 (green) in microglia (Iba-1, red) of the spinal dorsal horn in sham and CCI groups on day 5 after surgery. Images in white boxes are the amplification of an area in the corresponding image. Scale bar 100 m outside of the white frame and 25 m in the white frame. **b** Graphs showing the percentages of EZH2- or H3K27me3-labeled microglia of the spinal dorsal horn in the CCI and sham groups. Data are presented as the mean SEM (*n* = 6). ^*^ *p* < 0.05 vs. sham group. **c** Double-staining immunofluorescent images showing the expression of EZH2 (green) and H3K27me3 (green) in neurons (NeuN, red) of the spinal dorsal horn in the sham and CCI groups on day 5 after surgery. **d** Graphs showing the percentages of EZH2- or H3K27me3-labeled neurons in the spinal dorsal horn of the CCI and sham groups. Data are presented as the mean SEM (*n* = 6). *NS* no statistical difference. **e** Western blot analyses of EZH2 and H3K27me3 expression in the spinal dorsal horn on 0, 1, 3, 5, 7, 10 and 14 days after CCI surgery, respectively. The mean optic densities of the proteins were calculated by normalizing to GAPDH. All values are expressed as the means SEMs (*n* = 4).^*^p < 0.05 vs. sham group

To determine the role of EZH2-mediated H3K27me3 in the genesis of neuropathic pain, we examined EZH2 and H3K27me3 expression in the spinal dorsal horn ipsilateral to the CCI rats. In comparison with the sham group, the CCI group showed increased expression of EZH2 and H3K27me3 in microglia, especially in laminae III of the spinal cord on postoperative day 5 (Fig. 2a, b). Furthermore, immunofluorescent double staining showed that EZH2 and H3K27me3 are mainly expressed in neurons of the spinal dorsal horn in the sham group (Fig. 2c). The percentages of EZH2- and H3K27me3-labeled neurons in the spinal dorsal horn were also quantified and showed little-to-no difference between sham and CCI-treated rats (Fig. 2c, d).

Western blot data showed that CCI evoked significant increases both in EZH2 and H3K27me3 protein expressions on postoperative days 1, 3, 5, 7, 10, and 14 respectively (Fig. 2e). The largest increases in expressions for these time points were seen on day 3 for EZH2 and on day 5 for H3K27me3 post operation. The increased expressions for both molecules were still statistically evident in the groups on postoperative days 7, 10, and 14, but showed gradual recovery. EZH2 is a methyltransferase that catalyzes H3K27me3. CCI-induced the increase in expression of EZH2 occurred prior to an increase in H3K27me3 protein, suggesting that EZH2 may mediate H3K27me3 expression in the spinal dorsal cord of the CCI rat.

### CGRP8-37 and GSK126 prevented the development of neuropathic pain

**Fig. 3:**
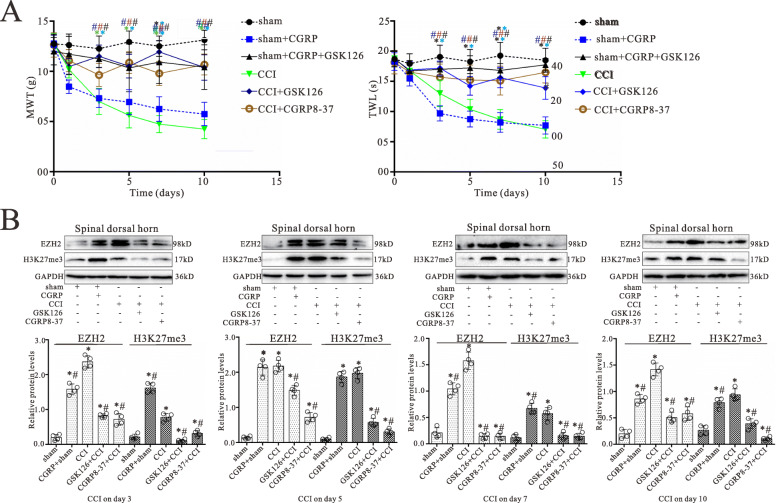
Intrathecal CGRP antagonist and EZH2 inhibitor administration prevent the pain hypersensitivity and attenuate increased levels of EZH2 and H3K27me3 in the spinal dorsal horn induced by CCI. **a** shows the mechanical withdrawal threshold (MWT) and thermal withdrawal latency (TWL) during the 10-day observation period in rats treated with daily intrathecal injection 1 M CGRP, 5 nM GSK126, 2 M CGRP8-37, or vehicle in 10 L for 9 days, respectively. All values are expressed as the means SEMs (*n* = 6). ^*^p < 0.05 vs. sham groups; ^#^*p* < 0.05 vs. CCI alone groups. **b** Western blot analyses for EZH2 and H3K27me3 protein levels in the spinal dorsal horn with CCI surgery for 3, 5, 7, and 10 days, respectively. Data were obtained from the spinal dorsal horn of animals treated with daily intrathecal injection 1 M CGRP, 5 nM GSK126, 2 M CGRP8-37, or vehicle in 10 L for 2, 4, 6, and 9 days, respectively. The mean optic densities of the proteins were calculated by normalizing to GAPDH. All values are expressed as the means SEMs (*n* = 4).^*^p < 0.05 vs. sham groups; ^#^*p* < 0.05 vs. CCI alone groups.

To determine the effects of CGRP and EZH2 on the development of chronic pain in CCI rats, we examined whether CGRP8-37 (a CGRP antagonist) and GSK126 (a potent, highly selective inhibitor of EZH2) can prevent the development of neuropathic pain. Rats were randomly assigned into six groups: sham, sham + CGRP, sham + CGRP + GSK126, CCI, CCI + GSK126, and CCI + CGRP8-37 groups. Behavior analyses were performed on day 1 before the surgery and then on postoperative days 1, 3, 5, 7, and 10, respectively. Rats in the sham + CGRP, CCI + GSK126, and CCI + CGRP8-37 groups received CGRP (1 M), GSK126 (5 nM), or CGRP8-37 (2 M) respectively, and the sham + CGRP + GSK126 group received both CGRP (1 M) and GSK126 (5 nM), in 10 L through the pre-implanted intrathecal catheter on day 1 immediately prior to the surgery and then daily till day 9 after the surgery. Vehicles (10 L) were administered to rats in the CCI and sham groups as controls. As shown in Fig. 3a, MWT and TWL in CCI and sham + CGRP groups were significantly lower than those of both in the sham groups on postoperative days 3 to 10 (*p* < 0.05; *n* = 6). Compared with CCI alone, MWT and TWL in the sham + CGRP + GSK126, CCI + GSK126, and CCI + CGRP8-37 groups were significantly higher than those of both in the CCI group on postoperative days 3 to 10 (*p* < 0.05; *n* = 6). These data demonstrate that CCI and CGRP treatment induced mechanical allodynia and thermal sensitivity of hind paws, whereas inhibition of CGRP and EZH2 can prevent the nerve injury-induced neuropathic pain.

### CGRP8-37 and GSK126 inhibited the CCI-induced increases of EZH2 and H3K27me3 in the spinal dorsal horn

Using the western blot technique, levels of EZH2 and H3K27me3 in the spinal dorsal horn were examined following treatment of GSK126 and CGRP8-37 with CCI rats. Animal grouping and treatment of CGRP, GSK126, and CGRP8-37 are the same as the animal behavioral tests described above. As shown in Fig. 3b, CCI and CGRP treatments significantly increased EZH2 and H3K27me3 protein expressions on postoperative days 3, 5, 7, and 10, respectively (*p* < 0.05; *n* = 4). Compared with CCI alone, CCI with GSK126 and CGRP8-37 markedly reversed the CCI-induced increase of both EZH2 and H3K27me3 protein expressions (Fig. 3b). Thus, it appears that CGRP may mediate CCI-induced EZH2 increase and subsequently increase in H3K27me3 protein in the spinal dorsal horn following nerve injury.

### CGRP increases EZH2 and H3K27me3 expressions in microglia by PKA/PKC

**Fig. 4.**
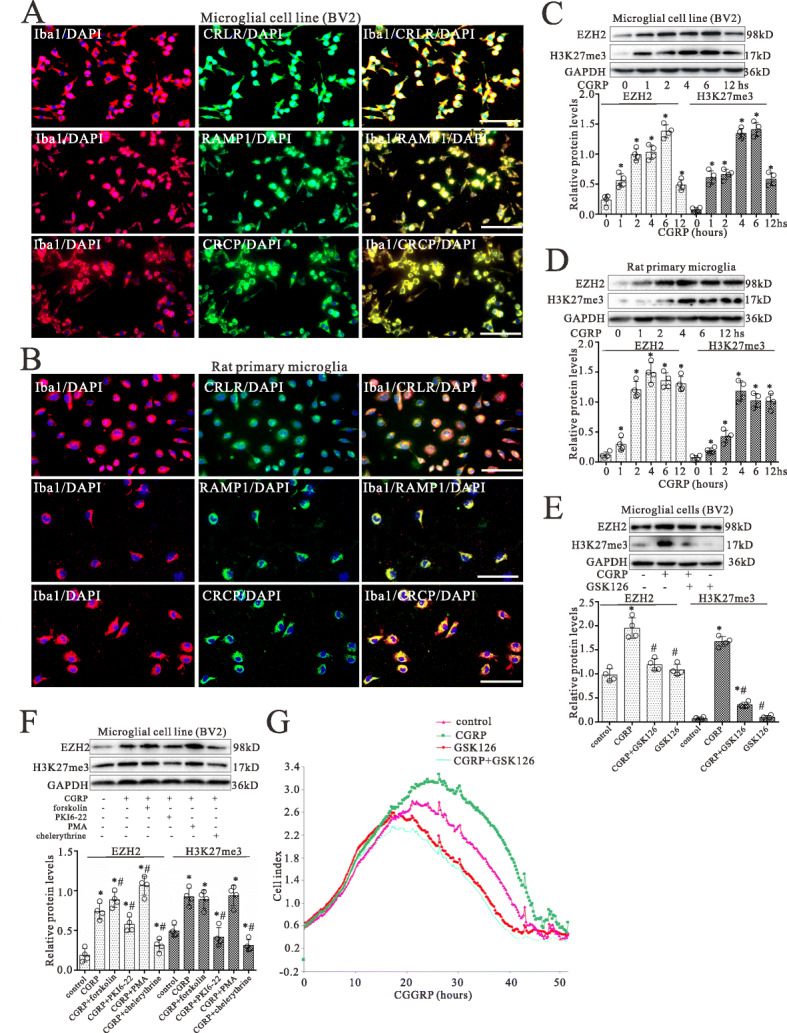
CGRP evokes increases in the expressions of EZH2 and H3K27me3 in microglia by PKA/PKC. **a**, **b** The expression of Iba1 (a marker of microglia, red) and its colocalization with CRLR, RAMP1, or CRCP staining (green) in cultured BV2 cells **a** and rat primary microglia **b**. Scale bar 40 m. **c**, **d** Western blot analyses of EZH2 and H3K27me3 expressions in BV2 cells **c** and rat primary microglia **d** with treatment of CGRP at 0, 1, 2, 4, 6, and 12 h, respectively. **e** Western blot analyses for EZH2 and H3K27me3 protein levels in BV2 microglial cells with co-treatment of CGRP (1 M) and GSK126 (5 nM) for 4 h. **f** Western blotting analyses for EZH2 and H3K27me3 protein levels in microglial cells (BV2) with treatment of CGRP peptide (1 M) for 4 h and pretreatment with 1 M forskolin (PKA activator), 3 M PKI6-22 (PKA inhibitor), 325 nM PMA (PKC activator), or 5 M chelerythrine chloride (PKC inhibitor) for 30 min. The mean optic densities of the proteins were calculated by normalizing to GAPDH. All values are expressed as the means SEMs (*n* = 4).^*^p < 0.05 vs. controls, ^#^*p* < 0.05 vs. CGRP only groups. **g** Shown is an example of microglial cell growth curves by RTCA. RTCA was performed to evaluate the proliferation and viability of microglial cells with continuous treatment of CGRP and co-treatment of GSK126

To study the effect of CGRP on microglia, we first investigated the expression of CGRP receptor components on microglia. Figure 4a and b show examples of co-expression of CRLR, RAMP1, and CRCP with the Iba1 staining on BV2 cells and rat primary microglia in culture. Nearly all of the Iba1-positive microglial cells expressed CGRP receptor components CRLR, RAMP1, and CRCP immunoreactivity.

The expression of both EZH2 and H3K27me3 in microglia was assessed by western blot following treatment with CGRP for 0, 1, 2, 4, 6, and 12 h, respectively. As shown in Fig. 4c and d, treatment with CGRP significantly increased the expression of both EZH2 and H3K27me3 protein levels in microglia (*p* < 0.05; *n* = 4). CGRP was found to induce the expression of both EZH2 and H3K27me3 in a time-dependent manner with a maximal effect observed after CGRP treatment for 46 h. However, CGRP with GSK126 partially or completely blocked the increased effect of CGRP on the increases of EZH2 and H3K27me3 expression (Fig. 4e).

Compared with CGRP alone, CGRP with forskolin and PMA (PKA and PKC activators) increased EZH2 into much higher levels and had the same effect on H3K27me3 as CGRP alone, and in turn PKI6-22 and chelerythrine chloride (PKA and PKC inhibitors) partially or completely blocked the increased effect of CGRP on EZH2 and H3K27me3 expressions (Fig. 4f).

### CGRP promoted the proliferation and viability of microglial cells

In order to determine the effect of CGRP on microglial cells, the cell proliferation and viability were assessed using RTCA following treatment of microglial cells with CGRP. RTCA proliferation assay demonstrated that the cell index increased in a time-dependent manner following CGRP treatment and was significantly higher in the CGRP group when compared with the control group following treatment after 24-h treatment (Fig. 4g). Compared with CGRP alone, CGRP with GSK126 completely blocked the increased effect of CGRP on cell proliferation and viability after 24-h treatment (*p* < 0.05; *n* = 3).

### Genome-wide profile of H3K27me3 enrichments in microglia after CGRP treatment

To investigate the role of H3K27me3 on microglia after treatment with CGRP, the profile of H3K27me3 enrichments in the microglial cell line (BV2) was analyzed using an Illumina HiSeq 4000 sequencing technique after stimulation with CGRP for 4 h. MACS v1.4.2 (Model-based analysis of ChIP-seq) software was used to detect the ChIP-enriched regions (peaks) from ChIP-seq data. The differentially enriched regions with statistical significance between the CGRP-treated group and control were identified by diffReps (Detecting Differential Chromatin Modification Sites from ChIP-seq Data with Biological Replicates, Cutoff: FC = 2.0, *p* = 0.0001).

**Fig. 5:**
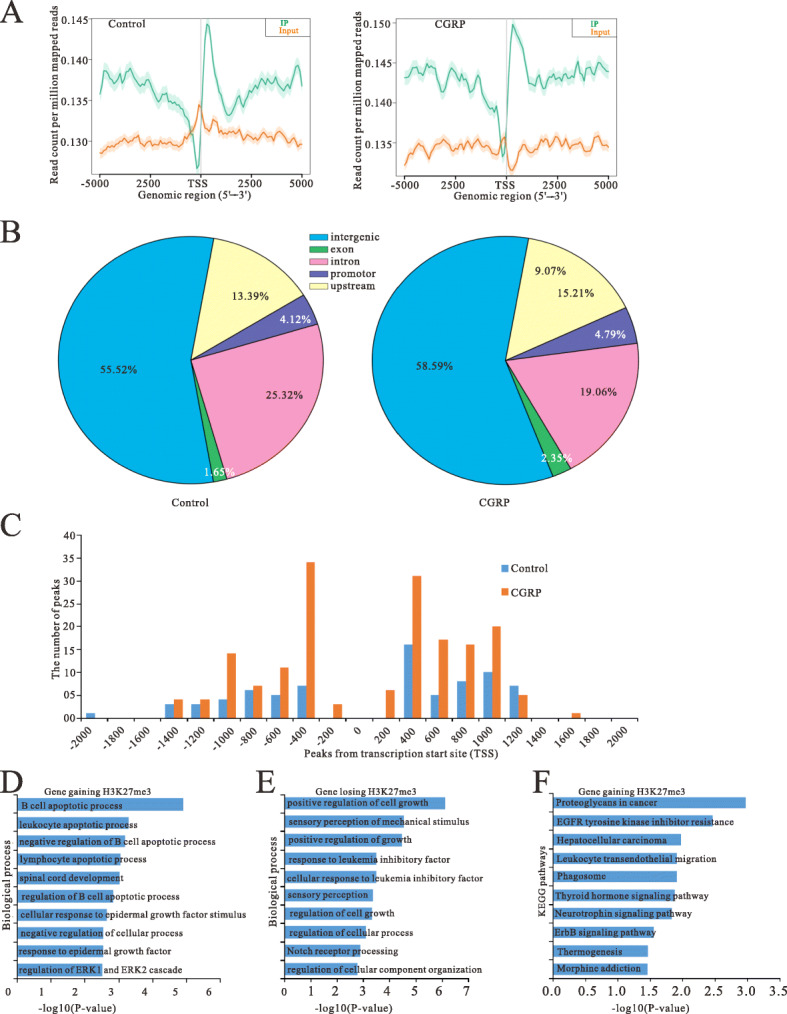
Effect of CGRP on the peak distribution of the ChIP-seq reads of H3K27me3 in microglial cells treated with CGRP compared with controls. **a** Metagene representation of average enrichment profiles of H3K27me3 in microglial cells treated with CGRP and controls. The *y* axis represents the numbers of the total sites that were identified as H3K27me3 peaks. **b** The distribution of CGRP-mediated H3K27me3 peaks relative to annotated genes in miroglial cells treated with CGRP and controls. **c** The distribution of H3K27me3 enrichment peaks on promoters relative to gene transcription start sites (TSSs). Shown is H3K27me3 peak frequencies relative to the distance from the nearest annotated TSS in microglial cells treated with CGRP and controls. **d**, **e** GO annotation of genes gaining H3K27me3 **d** and genes losing this mark **e** of CGRP treatment group vs. control. Bar plots show the top 10 enrichment values of the significant enrichment terms involving biological process (BP). **f** KEGG pathway analysis of genes gaining H3K27me3 in microglial cell treatment with CGRP. The bar plot shows the top 10 enrichment values of the significant enrichment terms involving KEGG pathways

Average H3K27me3 profiles are similar in control and CGRP-treated cells (Fig. 5a). A strong enrichment of H3K27me3 occurs from 2000 to + 2000 bp across the TSSs, including many sites located in downstream proximal regions of TSSs in CGRP-treated microglia or controls (Fig. 5a), corresponding to the position of the nucleosome-depleted region [24]. However, there were substantial alterations in the numbers of H3K27me3-enriched genes in CGRP-treated cells, compared with controls (Fig. 5b). We identified a total of 248 gene promoters, whose H3K27me3 enrichments are significantly altered in microglia treated with CGRP, including 173 gaining H3K27me3 (Supplementary Table S2), and 75 losing this mark (Supplementary Table S3), compared with controls. The distribution of H3K27me3-enriched promoters was mapped to proximal regions of TSSs of RefSeq genes (Fig. 5c).

### GO analysis of peaks relative to annotated genes

To further understand the function of annotated genes related to peaks, they were functionally classified using GO terminology. According to the functional annotation in GO database, gene promoters gaining H3K27me3 were mostly enriched for biological process (BP) terms associated with B cell apoptotic process (TRAF3IP2, BCL2L11, ITGAM), response to epidermal growth factor (SUPT4H1, ALYREF), and regulation of ERK1 and ERK2 cascade (DAB2, NLRP12, ARAF, DDT) (Fig. 5d, Supplementary Table S4).

Meanwhile, gene promoters losing H3K27me3 were enriched in BP terms such as positive regulation of cell growth (WNT3, ADAM10), response to leukemia inhibitory factor (MIR467A-2, MIR467A-4) and Notch receptor processing (ADAM10, PSEN2) (Fig. 5e, Supplementary Table S5).

### KEGG pathway analysis of peaks relative to annotated genes

KEGG pathway enrichment analysis was performed using the software KOBAS. The *p*< 0.05 was set as the threshold of significant enrichment. Based on the KEGG pathway analysis, gene promoters gaining H3K27me3 were significantly enriched in 13 pathways, including EGFR tyrosine kinase inhibitor resistance (ARAF, BCL2L11, GAB1, PLCG2), leukocyte transendothelial migration (ACTB, ITGAM, JAM2, PLCG2) and phagosome (ACTB, H2-M1, H2-M5, ITGAM, MSR1) (Fig. 5f, Supplementary Table S6). However, gene promoters losing H3K27me3 were significantly enriched in one pathway, the apelin signaling pathway (NRF1, PLIN1, SLC8A3).

### CGRP altered the gene expression in microglial cells associated with microglial activation

**Fig. 6:**
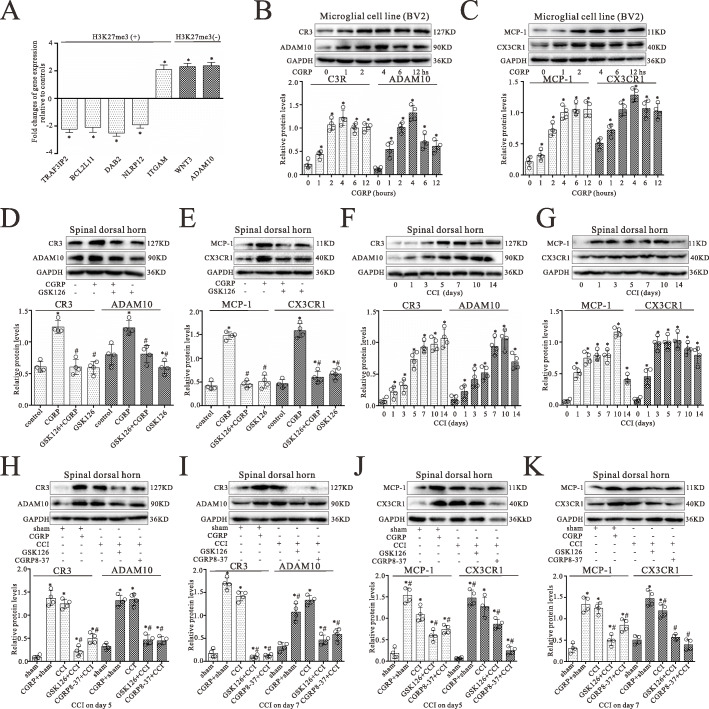
CGRP altered the gene expression in microglial cells associated with microglial activation. **a** Quantitative RT-PCR analysis for differences in expression levels of H3K27me specific target genes between CGRP-treated microglial cells and controls in the subset of genes gaining or losing H3K27me3 on their promoters. Results were calculated by normalizing to GAPDH in the same sample with the Ct method. Changes in relative levels of gene mRNAs expressed as folds of controls. All values were mean SEM. ^*^p < 0.05 (*n* = 3). **b**, **c** Western blot analyses of ITGAM (CR3) and ADAM10 **b** or MCP-1 and CX3CR1 **c** expressions in microglial cells (BV2) with treatment of CGRP at 0, 1, 2, 4, 6, and 12 h, respectively. **d**, **e** Western blotting analyses for ITGAM (CR3) and ADAM10 **d** or MCP-1 and CX3CR1 **e** protein levels in microglial cells (BV2) with co-treatment of CGRP (1 M) and GSK126 (5 nM) for 4 h. **f**, **g** Western blot analyses of ITGAM (CR3) and ADAM10 **f** or MCP-1 and CX3CR1 **g** expressions in the spinal dorsal horn on 0, 1, 3, 5, 7, 10, and 14 days after CCI surgery, respectively. **hk** Western blot analyses of ITGAM (CR3) and ADAM10 **h**, **i** or MCP-1 and CX3CR1 **j**, **k** expressions in the spinal dorsal horn with CCI surgery for 5 and 7 days, respectively. Data were obtained from the spinal dorsal horn of animals treated with daily intrathecal injection of either 1 M CGRP (10 L), 2 M CGRP8-37(10 L), 5 nM GSK126 (10 L), or vehicle (10 L) for 4 and 6 days, respectively. The mean optic densities of the proteins were calculated by normalizing to GAPDH. All values are expressed as the means SEMs (*n* = 4).^*^p < 0.05 vs. sham groups; ^#^*p* < 0.05 vs. CCI only groups

Since H3K27me3 was a repressive marker for gene expression, we next addressed the impact of gain or loss of H3K27me3 induced by CGRP on gene expression. We selected a subset of genes annotated with GO terms enriched among genes gaining or losing H3K27me3 (TRAF3IP2, BCL2L11, ITGAM, DAB2, NLRP12, WNT3, ADAM10) and assessed their expression in microglia after treatment of CGRP for 4 h by qRT-PCR. Results showed that most of genes gaining H3K27me3 became significantly downregulated, and genes losing this mark were significantly upregulated compared with controls (Fig. 6a). However, altered H3K27me3 on promoters did not have a pronounced effect on some gene expression (e.g., ITGAM). Candidate genes ITGAM (CR3) and ADAM10 play important roles in microglial activation. CX3CR1 and MCP-1 have been demonstrated to be associated with microglia/macrophage activation through EZH2 [8, 25]. Therefore, these four molecules were selected, and their expressions in microglia were examined by western blot. As shown in Fig. 6b and c, CGRP significantly increased ITGAM, ADAM10, MCP-1, and CX3CR1 protein levels in microglia following CGRP treatment, whereas EZH2 inhibitor partially or completely blocked these CGRP increase effects (Fig. 6d, e).

Furthermore, western blot results showed that CCI or CGRP treatment significantly increased ITGAM (CR3), ADAM10, MCP-1, and CX3CR1 protein levels in the spinal dorsal horn, compared with the sham groups (*p* < 0.05; *n* = 4) (Fig. 6f, g). However, CCI with GSK126 and CGRP8-37 markedly reversed the CCI-induced the increase of ITGAM (CR3), ADAM10, MCP-1, and CX3CR1 protein expressions on postoperative days 5 and 7, respectively (Fig. 6h-k).

## Discussion

The present study was to examine the facilitating nociceptive effect and possible mechanism of CGRP in the CCI rat model. We demonstrated that CGRP was able to upregulate EZH2-mediated H3K27me3 protein levels through PKA/PKC pathways in microglia. ChIP-seq data indicated that treatment of CGRP with microglia remarkably altered enrichments of H3K27me3 on gene promoters that were mostly associated with microglial activation, proliferation, and inflammation. We found that the CGRP antagonist suppressed the increases of ITGAM, ADAM10, MCP-1, and CX3CR1 expressions, key mediators of microglial activation, and the development of neuropathic pain via EZH2 in CCI rats. Our findings highly indicate that CGRP is implicated in the genesis of neuropathic pain through regulating microglial activation via EZH2-mediated H3K27me3 in the spinal dorsal horn following nerve injury.

CGRP has been implicated in the processing of nociceptive information in the spinal cord, which involves increased neuronglia interactions [18]. Accumulating evidence showed that CGRP receptors present in most of the dorsal horn neurons and co-localize with AMPA receptor [20]. Under an inflammatory condition, microglia expressed the CGRP receptor subunit RAMP1, which confers selectivity for CGRP and CGRP8-37 [10, 11, 26]. Previous study showed that the release of CGRP from terminals of afferents in the dorsal horn might not only facilitate glutamate-driven neuronal nociceptive signaling, but also act on glial CGRP receptors and lead to release ATP following nerve injury [9, 18, 19]. In the present work, we showed that CGRP immunoreactive levels were significantly correlated with Iba1 expression in the dorsal horn in CCI rats. Some CGRP immunostained fibers were found to closely approach and surround Iba1 immunopositive microglia. Importantly, CGRP promoted the microglial activation and proliferation following treatment of CGRP. Because of the vicinity of these structures to the CGRP-immunoreactive fibers, we hypothesize that activation of microglia in the spinal dorsal cord depend on the release of CGRP from fibers to induce microglial activation [9,11]. These data suggested that spinal microglia might be activated by CGRP released from CGRP containing fibers after CCI, which is increased in CGRP-positive terminals. Therefore, CGRP release from afferent terminals might be critically involved in the initiation and maintenance of microglial activation in the spinal dorsal horn.

Accumulating evidence has demonstrated that epigenetic mechanisms play an indispensable role in the regulation of glial function, specifically, in the control of microglial activation during neuroinflammation [6, 27]. EZH2 signals are increased in a group of proinflammatory cytokine genes that are upregulated in glial cells and involved in microglial proliferation [6]. Our results showed that EZH2 and H3K27me3 were mainly expressed in the neurons of the spinal dorsal horn in the sham group but obviously increased in the number of microglia in the CCI group. Because the increase of CGRP expression was accompanied by overexpression of EZH2 and H3K27me3 in microglia of the spinal dorsal horn, with little-to-no change in neurons of CCI rats, it is possible that CGRP induces H3K27me3 by EZH2 and that this links to the activation of microglia after nerve injury. Consistent with this hypothesis, a previous report showed that EZH2 was predominantly expressed in neurons of the spinal dorsal horn under normal conditions, and nerve injury drastically increased the number of microglia with EZH2 expression by more than 7 fold in the spinal dorsal horn [8]. Furthermore, we found that CGRP increased EZH2 and H3K27me3 levels in the spinal dorsal horn and in cultured microglia, but intrathecal injection of CGRP antagonist and EZH2 inhibitor decreased EZH2 and H3K27me3 levels in the spinal cord of CCI rats and suppressed the CGRP- and CCI-induced neuropathic pain. Thus, in the spinal cord, the increased release of sensory neuron-derived CGRP may activate CGRP receptors expressed on microglia leading to up-regulation of EZH2/H3K27me3 of which can mediate inflammatory gene expression, thereby facilitating nociception in CCI rats [8, 19, 20].

H3K27me3 modifications are traditionally known to be a repressive mark and are generally associated with silenced promoters. In order to obtain insights into the H3K27me3 target gene function, we mapped H3K27me32 enrichment profiles induced by CGRP at these loci using ChIP-seq in mouse microglial cells following CGRP treatment. Bioinformatics analysis showed that H3K27me3 enrichments on gene promoters in microglia treated with CGRP were mainly associated with cell proliferation, phagosome, and inflammation. Consistent with the ChIP-Seq results, the expression of key genes was confirmed in microglial cells treated with CGRP (TRAF3IP2, BCL2L11, ITGAM, DAB2, NLRP12, WNT3, ADAM10). Most of these genes have been previously reported in the regulation of microglial proliferation and activation, pro-inflammatory cytokine production, and neuroinflammation [28,31]. Association with microglial activation- and proinflammatory cytokine-related genes seems therefore to be a feature of CGRP mediating the altered H3K27me3 enrichments on the gene promoters in microglia.

Among identified candidate genes gaining H3K27me3, TRAF3IP2 and BCL2L11 are apoptotic genes and play a promoting role in the apoptosis in glial cells [32, 33]. Increased enrichment of H3K27me3 on TRAF3IP2 and BCL2L11 gene promoters may promote microglial proliferation. Among candidate genes losing this repressive mark, WNT3 overexpression in the dorsal horn leads to the activation of microglia, then triggers BDNF secretion that is responsible for the establishment of neuropathic pain [34]. ADAM10 that cleaves CX3CL1 into a secreted form is involved in microglial activation and microglia-mediated neuroinflammation in the spinal dorsal horn following nerve injury [35]. CX3CR1, a microglia-specific receptor for CX3CL1, may play a crucial role in regulation of phagocytosis and inflammatory cytokines in microglial activation via the p38MAPK/PKC pathway [36]. Furthermore, ADAM10 influences the function of MCP-1, the novel target of ADAM10 upon inflammation and immune cell recruitment [37] and microglial activation [38]. ITGAM (CR3) is a microglial cell biomarker and associated with spinal microglial activation induced by peripheral nerve injury [39]. Previous report demonstrated that microglial activation mediated early synapse elimination via both phagocytic signaling through ITGAM (CR3) and chemokine signaling through CX3CR1 in mouse models of neurodegeneration [40], suggesting a functional interaction between microglial activation and synaptic plasticity following injury. In the present study, we found that CGRP increased in the expressions of ITGAM (CR3) and CX3CR1 in the spinal dorsal horn and cultured microglial cells; CGRP antagonists inhibited these increase induced by CCI. Therefore, it is possible that CGRP-acting microglia mediates CCI-induced neuropathic pain through microgliasynapse interactions via ITGAM (CR3) and CX3CR1 signals [41]. Despite MCP-1 being mainly released by injured neurons, microglia also express MCP-1 under inflammation condition [42, 43], consistent with our results that MCP-1 was expressed in cultured microglia by EZH2 following CGRP treatment. Our results showed that CGRP increased the protein levels of ADAM10, CR3, CX3CR1, and MCP-1 in the spinal dorsal horn and cultured microglia through EZH2, suggesting that the increased EZH2/H3K27me3 expression by CGRP might be contributed to the microglial activation and its production of inflammatory mediators, which associated with local neuroinflammation in the spinal cord. Furthermore, our results showed that CGRP could increase H3K27me3 enrichment on the genes of TRAF3IP2, BCL2L11, and ITGAM (CR3) and attenuate this mark on the genes of WNT3 and ADAM10; these might contribute to microglia proliferation, activation, and production of proinflammatory mediators by the redistribution of H3K27me3 in microglia [44]. A previous study showed that the expression of EZH2 globally increased the abundance of H3K27me3 induced both repression and activation of polycomb-regulated loci [45], similar to our results. Moreover, we identified that microglial H3K27me3 or EZH2, rather than functioning as a repressor, mediate CGRP-induced proinflammatory gene expression, and therefore EH2 inhibitor or CGRP antagonist diminishes microglial activation and attenuates the development of allodynia in rats with CCI-induced neuropathic pain.

## Conclusion

In summary, our current study reveals that CGRP plays a critical role in the development of neuropathic pain through regulating the microglial activation via EZH2-mediated H3K27me3 in microglia. Genomic analyses suggested that genes with the redistribution of H3K27me3 induced by CGRP are involved in microglial activation and inflammation-related gene expression that might be associated with neuropathic pain. ITGAM, ADAM10, MCP-1, and CX3CR1, key mediators of microglial activation, were identified in the CCI rat model and might be crucial in the development of neuropathic pain. These results could give us a clue to new therapeutic targets for treatment of neuropathic pain. However, further studies are needed to confirm our results.

## Supplementary Information

**Additional file 1: Supplementary Table S1.** Gene specific primer sequences used in the study.

**Additional file 2: Supplementary Table S2.** Gene promoters gaining H3K27me3 in CGRP treated group.

**Additional file 3: Supplementary Table S3.** Gene promoters losing H3K27me3 in CGRP treated group.

**Additional file 4: Supplementary Table S4.** Biological processes (BP) result of genes gaining H3K27me3 in CGRP treated group.

**Additional file 5: Supplementary Table S5.** Biological processes (BP) result of genes losing H3K27me3 in CGRP treated group.

**Additional file 6: Supplementary Table S6.** Kyoto Encyclopedia of Genes and Genomes (KEGG) result of genes gaining H3K27me3 in CGRP treated group.

## Data Availability

The key data are included in the figures, tables, and additional files. The full datasets that were analyzed are available from the corresponding author on reasonable request.

## Abbreviations

BP: Biological processes
CCI: Chronic constriction injury
CGRP: Calcitonin gene-related peptide
CRCP: Receptor component protein
CRLR: Calcitonin receptor-like receptor
ChIP-seq: Chromatin immunoprecipitation sequencing
EZH2: Enhancer of zeste homolog-2
GO: Gene Ontology
H3K27me3: Histone H3 lysine 27 trimethylation
KEGG: Kyoto Encyclopedia of Genes and Genomes
RAMP1: Receptor activity-modifying protein 1
RTCA: Real-time cell analysis
TSSs: Transcription start sites
