# Supplementary material for: Calcitonin gene-related peptide regulates spinal microglial activation through the histone H3 lysine 27 trimethylation via enhancer of zeste homolog-2 in rats with neuropathic pain
Source: J Neuroinflammation. 2021 May 21;18:117. doi: 10.1186/s12974-021-02168-1 (PMC8139106; doi:10.1186/s12974-021-02168-1)
Supplement: Supplementary file 1 — Additional file 1: Supplementary Table S1. Gene specific primer sequences used in the study. [file 12974_2021_2168_MOESM1_ESM.docx]

**Supplementary Table S1: Gene specific primer sequences used in the study.**

| Genes | primer sequence | annealing (℃) | length (bp) |
| --- | --- | --- | --- |
| GAPDH | F:5’ CACTGAGCAAGAGAGGCCCTAT3’  R:5’ GCAGCGAACTTTATTGATGGTATT3’ | 60 | 144 |
| BCL2L11 | F:5’ AAATGGCCAAGCAACCTTCTG3’  R:5’ TGTCCACCTTCTCTGTCACAC3’ | 60 | 55 |
| Itgam | F:5’ CAGGGCAGGAGTCGTATGTG3’  R:5’ GTCCATCAGCTTCGGTGTTG3’ | 60 | 289 |
| TRAF3IP2 | F:5’ AGCCCGAGGAACTCTAAGAAC3’  R:5’ GGGGCTGATTGCTACGATTA3’ | 60 | 235 |
| Dab2 | F:5’ GCTAATCGGTATTGATGATGTGC3’  R:5’ TGCTGCCATTCCCTTGAGTT3’ | 60 | 85 |
| NLRP12 | F:5’ CCTTCGGTTGGGCATTTG3’  R:5’ TTGGAGGTGAGTCCGCAGTT3’ | 60 | 204 |
| Wnt3 | F:5’ TCTTCCACTGGTGCTGCTATGT3’  R:5’ TGGGTCCAGGTCGTTTATCAC3’ | 60 | 225 |
| Adam10 | F:5’ AACGGGATGGTGGAACAAG3’  R:5’ AAAGTTGGGCTTGGGATCA3’ | 60 | 273 |
